# Underweight is independently associated with mortality in post-operative and non-operative patients admitted to the intensive care unit: a retrospective study

**DOI:** 10.1186/1471-227X-4-3

**Published:** 2004-10-05

**Authors:** Javier D Finkielman, Ognjen Gajic, Bekele Afessa

**Affiliations:** 1 Division of Pulmonary and Critical Care Medicine, Department of Internal Medicine, Mayo Clinic College of Medicine, Rochester, MN, USA

## Abstract

**Background:**

Low and high body mass index (BMI) have been recently shown to be associated with increased and decreased mortality after ICU admission, respectively. The objective of this study was to determine the impact of BMI on mortality and length of stay in patients admitted to the intensive care unit (ICU).

**Methods:**

In this retrospective cohort study, the Acute Physiology and Chronic Health Evaluation (APACHE) III database of patients admitted to the ICUs of a tertiary academic medical center, from January 1997 to September 2002, was crossed with a Hospital Rule-based Systems database to obtain the height and weight of the patients on admission to the ICU. The cohort was divided in post-operative and non-operative groups. We created the following five subgroups based on the BMI: <18.5, 18.5 to 24.9, 25 to 29.9, 30.0 to 39.9, ≥ 40.0 Kg/m^2^. A multiple logistic regression analysis was used to determine the independent impact of BMI on hospital mortality. The ICU length of stay ratio was defined as the ratio of the observed to the predicted LOS. *P*-value < 0.05 was considered significant. The 95% confidence interval (CI) was calculated for the odds ratio (OR).

**Results:**

BMI was available in 19,669 of the 21,790 patients in the APACHE III database; 11,215 (57%) of the patients were admitted post-operatively. BMI < 18.5 was associated with increased mortality in both post-operative (OR = 2.14, 95% CI, 1.39 to 3.28) and non-operative (OR = 1.51, 95% CI, 1.13 to 2.01) patients. Post-operative patients with a BMI between 30.0 to 39.9 had a lower mortality rate (OR = 0.68, 95% CI, 0.49 to 0.94). Post-operative patients with BMI <18.5 or BMI ≥ 40 had an ICU length of stay ratio significantly higher than patients with BMI between 18.5 to 24.9. The addition of BMI < 18.5 did not improve significantly the accuracy of our prognostic model in predicting hospital mortality.

**Conclusions:**

Low BMI is associated with higher mortality in both post- and non-operative patients admitted to the ICU. LOS is increased in post-operative patients with low and high BMIs.

## Background

The body mass index (BMI) is an anthropometric measure of nutritional status that is calculated as the weight in kilograms divided by the square of the height in meters[1]. The relationship between BMI and mortality has been shown to be J- or U-shaped in large population studies; the highest mortality was observed in persons with low and high BMIs [2-5]. Previous studies have shown that low BMI (but not high) is an independent predictor for mortality in patients admitted to the hospital[1,6,7]. Two recent studies have investigated the impact of BMI on ICU outcome. In a large retrospective study, Tremblay et al. found that low BMI (< 20 Kg/m^2^), but not high, was associated with increased mortality following admission to the ICU; the increased mortality was seen in medical and emergency surgical groups but not in the elective surgical group and the length of stay (LOS) was longer in severely obese and underweight patients[8]. In the prospective study by Garrouste-Orgeas et al., a low BMI (<18.5) was found to be associated with higher mortality and high BMI (>30 Kg/m^2^) with lower mortality[9].

Previous studies did not analyze the data from post-operative and non-operative patients separately when they looked at the impact of BMI on the outcome of critically ill patients. We undertook this study to determine the influence of BMI on mortality in post-operative and non-operative patients admitted to the ICU, in a single tertiary academic medical center. Based on the association of low BMI with increased mortality, a recent publication has highlighted the importance of looking at whether adding BMI would improve the predictive accuracy of the ICU prognostic models[9]. In the current study, we tested the hypothesis that adding BMI improves the predictive accuracy of the APACHE III prognostic system.

## Methods

In this retrospective, cohort study, we crossed the prospectively collected Acute Physiology and Chronic Health Evaluation (APACHE) III database of adult patients consecutively admitted to the intensive care units of Mayo Medical Center, Rochester, Minnesota, between January 1997 and September 2002, with a Hospital Rule-based Systems database that records the height and weight on admission to the ICU. Patients were admitted to one medical, two surgical and one multi-specialty ICU. Those patients admitted to the neurological, cardiovascular surgery, and coronary care units were not included since they were not part of the APACHE III database. Only first admissions were included in this study. The Mayo Foundation Institutional Review Board approved the study, and a waiver of informed consent was granted. Patients who did not authorize their medical records to be reviewed for research, and those whose weight or height values were missing were excluded.

Mayo Medical Center includes two hospitals with a total of approximately 1900 beds. The medical ICU is a 15-bed closed unit in Saint Mary's Hospital. The surgical ICUs consist of a general surgical/trauma 24-bed unit and a 20-bed surgical unit mainly for thoracic and vascular surgery patients, both located at Saint Mary's Hospital. The 12-bed (increased to 17 beds in March 2000) multi-specialty ICU is located in Rochester Methodist Hospital. The patient population in the multi-specialty ICU included liver, kidney, pancreas and bone marrow transplant recipients; and hematology, oncology, general surgery and orthopedic patients.

Data were obtained from the APACHE III database using the software provided by Cerner Corporation (Kansas City, MO). Data collected included age, ethnicity, gender, ICU admission source, admission type (postoperative or non-operative), intensity of treatment (low-risk monitor, high-risk monitor, active treatment), ICU admission diagnosis group, ICU length of stay (LOS), APACHE III score, APACHE III-predicted hospital mortality, APACHE III predicted-ICU LOS and hospital discharge status. The admission source was classified as operating room/recovery room (OR/RR), emergency room/direct admission from outpatient clinic (ER/direct), transfer from other floors of the same hospital and transfer from other institutions. All ICU admissions were categorized into three groups based on the intensity of treatment: "active treatment" if a patient received one or more of 33 items of the Therapeutic Intervention Scoring System (TISS) defined as ICU specific therapy on the first ICU day; "high-risk monitor" if a patient who did not receive active treatment on the first ICU day had greater than 10% probability of receiving active treatment during the ICU stay; and "low-risk monitor" if a patient who did not receive active treatment on the first ICU day had less than 10% probability of receiving active treatment during the ICU stay [10-12]. The ICU admission diagnosis groups included cardiovascular, genitourinary, gastrointestinal, hematologic, metabolic/endocrine, musculoskeletal/skin, neurologic, respiratory, transplant and trauma. The APACHE III score and predicted hospital mortality rate for each patient were calculated as described by Knaus and colleagues[13]. The type of ICU (medical, surgical or multi-specialty) to which each patient was admitted was recorded.

The body mass index for each patient (BMI) was calculated as the weight (in kilograms) divided by the square of the height (in meters). All BMIs are presented in Kg/m^2^. Four BMI subgroups were created using the cut-off points of the World Health Organization (WHO): 18.5 to 24.9 (normal range), 25.0 to 29.9 (grade 1 overweight), 30.0 to 39.9 (grade 2 overweight), ≥ 40 (grade 3 overweight)[14]. A fifth subgroup for BMI < 18.5 (underweight) was added as in the study of Calle et al[3].

Descriptive data were summarized as mean (standard deviation), median (interquartile range) (IQR) or percentages. The cohort was divided into post-operative and non-operative patients. The primary analysis consisted of a comparison of hospital mortality for each BMI subgroup. A multiple logistic regression model consisting of hospital mortality as a dependent variable, BMI subgroup, APACHE III predicted mortality, admission source, and intensity of treatment as independent variables was created to adjust for potentially confounding variables that could affect hospital mortality. In the post-operative group the admission source was not included in the model since the source in all the patients was either the recovery room or the operating room. This logistic regression model was based on a previous analysis that identified the variables independently associated with hospital mortality[15]. We performed another logistic regression analysis by entering into the model BMI < 18.5, APACHE III predicted mortality, admission source, and intensity of treatment as independent variables and hospital mortality as dependent variable. We calculated the area under the receiver operating characteristic curve (AUC) to determine the performance of the logistic regression models with and without BMI < 18.5 in discriminating survivors from non-survivors[16]. Differences in mortality rates were expressed as odds' ratios (OR) for death with 95 % confidence intervals (CI) and corresponding *P*-values.

The ICU LOS ratio was defined as the ratio of the observed to the predicted LOS. LOS ratios less than one indicate stays shorter than predicted[17]. In comparing differences in ICU LOS ratios among the BMI subgroups, we performed the Kruskal-Wallis test first. If the Kruskal-Wallis test showed statistically significant difference among groups, we subjected the data to further analysis by the Mann-Whitney U test to identify the BMI subgroup that was significantly different from the normal BMI subgroup.

StatView 5.0 (SAS Institute, Cary, NC) and MedCalc 7.3 (Mariakerke, Belgium) computer softwares were used for statistical analyses. Patients with missing data were excluded from analysis involving the missing elements. A *P*-value < 0.05 was considered statistically significant.

## Results

Of the 21,790 patients with first ICU admissions during the study period, 19,669 had height and weight data. Their baseline characteristics are listed in Table 1. Fifty seven percent of the patients were post-operative. Most of the patients in both post- and non-operative groups were males, whites, and received active treatment during their first ICU day. The most common admission diagnoses were cardiovascular and respiratory for post- and non-operative groups, respectively. The most common BMI subgroup was 25.0–29.9 in post-operative patients and 18.5–24.9 in non-operative patients (Table 2).

**Table 1:** Characteristics of 19,669 patients admitted to the intensive care unit

**Variables**	**Post-Operative**	**Non-Operative**
N	11,215	8,454
BMI (Kg/m^2^) (SD)	28.4 (7.9)	27.5 (7.2)
Mean age (SD)	64.2 (15.9)	60.9 (19.1)
Male sex (%)	59.2	54.1
White ethnicity (%)	96.1	94.6
Median APACHE III (IQR)	39.0 (29.0–51.0)	48 (32.0–67.0)
Median predicted mortality % (IQR)	2.5 (1.2–5.7)	7.5 (2.3–22.7)
Admission source (%)		
RR/OR	99.9	0.0
ER/Direct	0.0	49.0
Same hospital transfer	0.1	45.2
Other hospital transfer	0.0	5.8
ICU type (%)		
Surgical	78.8	26.9
Medical	0.4	53.4
Multi-specialty	20.8	19.7
Treatment intensity (%)		
Active	51.6	56.0
High-risk monitor	1.4	19.1
Low-risk monitor	46.9	24.9
Admission diagnosis group (%)		
Cardiovascular	31.4	24.7
Gastrointestinal	24.3	20.1
Respiratory	14.8	28.9
Musculoskeletal	11.3	1.2
Genitourinary	8.1	3.1
Trauma	4.1	6.9
Neurology	1.2	9.8
Metabolic/endocrine	1.3	3.0
Transplant	3.3	0.1
Hematology	0.2	2.2

SD = Standard deviation; IQR = Interquartile range; ICU = Intensive care unit

**Table 2:** The body mass index subgroups of 19,669 patients admitted to the intensive care unit

**BMI Subgroup**	**Number of patients (%)**	
	**Post-Operative N = 11,215**	**Non-Operative N = 8,454**
< 18.5	384 (3.4)	428 (5.1)
18.5–24.9	3,461 (30.9)	2,945 (34.8)
25.0–29.9	3,878 (34.6)	2,692 (31.8)
30.0–39.9	2,718 (24.2)	1,947 (23.0)
≥ 40.0	774 (6.9)	442 (5.2)

In the overall study population, BMI < 18.5 was independently associated with increased mortality (OR = 1.71, 95% CI, 1.34 to 2.17; *P *< 0.0001). Among the ICU admission diagnoses, BMI < 18.5 was associated with increased adjusted mortality in cardiovascular, genitourinary and musculoskeletal groups (Table 3). BMI of 30 to 39.9 was associated with increased and decreased mortality in the neurology and respiratory groups respectively (Table 3).

**Table 3:** The BMI subgroups that are independently associated with hospital mortality in each admission diagnosis group using BMI of 18.5 to 24.9 as reference

**Admission diagnosis**	**BMI subgroup**	**Odds Ratio (95%CI)**	***P*-value**
Cardiovascular	< 18.5	2.84 (1.70–4.74)	< 0.001
Genitourinary	< 18.5	4.15 (1.21–14.25)	0.0236
Gastrointestinal	None		
Hematology	None		
Metabolism/endocrine	None		
Musculoskeletal	< 18.5	3.70 (1.20–11.38)	0.0227
Neurology	30–39.9	2.74 (1.29–5.81)	0.0086
Respiratory	30–39.9	0.72 (0.56–0.94)	0.0138
Transplant	None		
Trauma	None		

For post-operative patients, the crude hospital mortality rate was 3.3 %, and the median (IQR) ICU LOS and ICU LOS ratio were 1.04 (0.82–2.15) and 0.42 (0.25–0.77) days, respectively. After adjusting for confounding variables, postoperative patients with a BMI < 18.5 had a higher hospital mortality, and those with BMI of 30.0–39.9 had a lower hospital mortality rate (Table 4). The median observed and predicted ICU LOS and the ICU LOS ratios for each BMI subgroup of the post-operative patients are listed in Table 5. There were statistically significant differences in the ICU LOS ratio between the various BMI subgroups (*P *< 0.0001 by Kruskal-Wallis test). Compared to the normal BMI subgroup, the ICU LOS ratio was higher in the BMI < 18.5 (*P *= 0.0411 by Mann-Whitney U test) and BMI ≥ 40 (*P *< 0.0001 by Mann-Whitney U test).

**Table 4:** Multivariate logistic regression analysis assessing the association of hospital mortality with APACHE III predicted hospital mortality, intensity of treatment, and BMI subgroup in 11,215 post-operative patients

	**Odds Ratio (95% CI)**	***P*-value**
**BMI**		
< 18.5	2.14(1.39–3.28)	**0.0005**
18.5–24.9	1.00	
25.0–29.9	0.86 (0.66–1.13)	0.2752
30.0–39.9	0.68 (0.49–0.94)	**0.0186**
≥ 40.0	0.75 (0.45–1.26)	0.2771
**Predicted mortality**	1.066 (1.059–1.073)	**<0.0001**
**Intensity of treatment**		
Low-risk monitor	0.55 (0.42–0.71)	**<0.0001**
High-risk monitor	0.66 (0.30–1.47)	0.3111
Active	1.00	

**Table 5:** Observed and predicted length of ICU stay and ICU length of stay ratio for post-operative patients

	**Median (IQR) ICU Length of Stay**		
**BMI**	**Observed**	**Predicted**	**Ratio**
< 18.5	1.54 (0.85–3.02)	3.80 (2.97–4.73)	0.45 (0.25–0.86)
18.5–24.9	1.03 (0.81–2.08)	3.57 (2.67–4.50)	0.40 (0.24–0.73)
25.0–29.9	1.04 (0.82–2.17)	3.61 (2.68–4.50)	0.41 (0.24–0.77)
30.0–39.9	1.03 (0.81–2.07)	3.55 (2.58–4.49)	0.41 (0.25–0.76)
≥ 40.0	1.57 (0.84–2.54)	3.29 (1.68–4.30)	0.54 (0.30–0.96)

LOS = Length of stay; ICU = Intensive care unit; IQR = Interquartile range

For non-operative patients, the crude hospital mortality rate was 16.4%, and the median (IQR) ICU LOS and ICU LOS ratio were 1.68 (0.89–3.69) and 0.45 (0.25–0.89) days, respectively. After adjusting for confounding variables, the patients with a BMI < 18.5 had a higher hospital mortality rate (Table 6). There were no statistically significant differences in the ICU LOS ratio among the five BMI subgroups as assessed by Kruskal-Wallis test (*P *= 0.3414) (Tables 7).

**Table 6:** Multiple logistic regression analysis assessing the association of hospital mortality with APACHE III predicted hospital mortality, intensity of treatment, admission source, and BMI subgroup in 8,450 non-operative patients

	**Odds Ratio (95% CI)**	***P*-value**
**BMI**		
< 18.5	1.51 (1.13–2.01)	**0.0051**
18.5–24.9	1.00	
25.0–29.9	1.02 (0.87–1.20)	0.8022
30.0–39.9	0.98 (0.82–1.17)	0.7979
≥ 40.0	0.86 (0.63–1.20)	0.3967
**Predicted mortality**	1.045 (1.042–1.048)	**<0.0001**
**Intensity of treatment**		
Low-risk monitor	0.39 (0.31–0.51)	**<0.0001**
High-risk monitor	0.87 (0.73–1.03)	0.1033
Active	1.00	
**Admission source**		
Other hospital	0.99 (0.75–1.32)	0.9596
ER/Direct	1.00	
Same hospital	1.43 (1.24–1.65)	**<0.0001**

*Four patients were excluded from this analysis because of missing data. CI = Confidence interval; RR = recovery room; OR = operating room; ER = emergency room; Direct = direct admission from the outpatient clinic

**Table 7:** Observed and predicted length of ICU stay and ICU length of stay ratio for non-operative patients

	**Median (IQR) ICU Length of Stay**		
**BMI**	**Observed**	**Predicted**	**Ratio**
< 18.5	1.71 (0.87–3.80)	4.38 (2.82–6.15)	0.45 (0.24–0.87)
18.5–24.9	1.65 (0.87–3.62)	4.01 (2.70–5.82)	0.44 (0.25–0.90)
25.0–29.9	1.63 (0.90–3.58)	4.13 (2.71–5.83)	0.45 (0.25–0.87)
30.0–39.9	1.73 (0.91–3.71)	4.08 (2.83–5.87)	0.46 (0.25–0.91)
≥ 40.0	1.94 (0.92–4.34)	4.39 (3.05–6.25)	0.49 (0.28–0.93)

LOS = Length of stay; ICU = Intensive care unit; IQR = Interquartile range

In discriminating hospital survivors from non-survivors, the AUC (95% CI) of the model with BMI < 18.5 was 0.859 (0.854 to 0.864) and the AUC of the model without BMI < 18.5 was 0.858 (0.853 to 0.863) (*P *= 0.102).

## Discussion

The results of our retrospective study suggest that a BMI <18.5 is independently associated with higher mortality in post- and non-operative patients admitted to the ICU. We found no difference in the ICU LOS among the BMI subgroups in non-operative patients. In post-operative patients, the LOS was longer in patients with a BMI <18.5 or BMI ≥ 40. We also noted that the addition of BMI does not improve significantly the predictive accuracy of the prognostic model.

In this study, we wanted to determine the impact of BMI on mortality in post- and non-operative patients separately since patients admitted to the ICUs from hospital wards have higher mortality than patients admitted from the operating room, independent of disease severity[17,18]. In our cohort, both post-operative and non-operative groups had a higher mortality rate when their BMI was < 18.5. Interestingly, a BMI between 30.0–39.9 was associated with lower mortality in post-operative patients. Although previous studies had included both post-operative and non-operative patients, they had not looked at the influence of BMI on mortality in these two groups separately[8,9]. Low BMI has been associated with higher mortality rate in hospitalized patient in both the ICU [8,9] and non-ICU settings[1,6,7]. Studies addressing the association of high BMI in patients admitted to the ICU and the hospital have given conflicting results. In a retrospective study of 184 blunt trauma victims, Choban et al found that mortality in patients with a BMI >31 and <27 was 42.1 and 5 %, respectively[19]. In a more recent study of 117 morbidly obese patients (BMI= 51.3 ± 25.9) compared to 132 other patients (BMI= 27.6 ± 3.1) selected randomly by a computer in the medical ICU, their mortality was 30 and 17 %, respectively[20]. In contrast, our study was similar to the studies by Tremblay et al.[8] and Garrouste-Orgeas et al.[9] in showing that a high BMI was not associated with high mortality in both post- and non-operative patients. We also found that a BMI between 30.0–39.9 (grade 2 overweight) was associated with decreased mortality in post-operative patients. Although obesity has long been considered a potential risk factor for poor outcome from a variety of surgical procedures, the evidence suggests that obesity does not result in an increase in mortality[21,22]. Obesity, however, has been associated with an increase in perioperative complications, particularly wound problems, which could explain our finding of a longer LOS in post-operative patients with BMI ≥ 40. Additionally, in a recent study of patients receiving mechanical ventilation for acute lung injury, a BMI > 30 was not associated with increased mortality [23].

Our study has several limitations. It has been speculated that a low BMI is simply the consequence of a serious illness before the hospitalization that it is ultimately fatal[1,6]. We have not adjusted for previous weight loss because this information was not available. However, studies that have adjusted for weight loss before the admission found that the independent effect on mortality of low BMI was unchanged[1,6]. Additionally, depending on the balance between fluid intake and output, the weight on admission to the ICU could be different to the patient's real weight, and because of the study design, we cannot ascertain the accuracy of the height and weight measurements. Although the APACHE III data were collected prospectively, our study has a retrospective design. Since our analysis was limited to the variables available in the APACHE III database, other confounding factors that might have influenced outcomes may not have been included. Moreover, because our study reflects the experience of a single tertiary institution with a unique system of health care delivery and without ethnic diversity, the results cannot be extrapolated to other medical centers.

## Conclusions

This study shows that a BMI < 18.5 is independently associated with increased mortality in post- and non-operative patients admitted to the ICU; and in post-operative patients, the LOS was longer in patients with a BMI < 18.5 or BMI ≥ 40. We also found that the inclusion of BMI < 18.5 in a prognostic model does not improve the accuracy of mortality prediction.

## Competing interests

The authors declare that they have no competing interests.

## Authors' contributions

JDF participated in conception, design, acquisition of the data, analysis of the data, and drafting of the manuscript.

OG participated in analysis of the data, and critical revision of the manuscript.

BA participated in conception, design, analysis of the data, statistical analysis, critical revision of the manuscript, and supervision.

## Pre-publication history

The pre-publication history for this paper can be accessed here:


